# Socio-economic inequalities in C-reactive protein and fibrinogen across the adult age span: Findings from *Understanding Society*

**DOI:** 10.1038/s41598-017-02888-6

**Published:** 2017-06-01

**Authors:** Apostolos Davillas, Michaela Benzeval, Meena Kumari

**Affiliations:** 1 Institute for Social and Economic Research (ISER), University of Essex, Colchester, UK; 2 Department of Epidemiology and Public Health, University College London, London, UK

## Abstract

Systemic inflammation has been proposed as a physiological process linking socio-economic position (SEP) to health. We examined how SEP inequalities in inflammation –assessed using C-reactive protein (CRP) and fibrinogen– varied across the adult age span. Current (household income) and distal (education) markers of SEP were used. Data from 7,943 participants (aged 25+) of *Understanding Society* (wave 2, 1/2010-3/2012) were employed. We found that SEP inequalities in inflammation followed heterogeneous patterns by age, which differed by the inflammatory marker examined rather than by SEP measures. SEP inequalities in CRP emerged in 30s, increased up to mid-50s or early 60 s when they peaked and then decreased with age. SEP inequalities in fibrinogen decreased with age. Body mass index (BMI), smoking, physical activity and healthy diet explained part, but not all, of the SEP inequalities in inflammation; in general, BMI exerted the largest attenuation. Cumulative advantage theories and those considering age as a leveler for the accumulation of health and economic advantages across the life-span should be dynamically integrated to better understand the observed heterogeneity in SEP differences in health across the lifespan. The attenuating roles of health-related lifestyle indicators suggest that targeting health promotion policies may help reduce SEP inequalities in health.

## Introduction

Although it is well established that socio-economically disadvantaged groups experience poor health, less is known about how the association between socio-economic position (SEP) and health evolves over the lifecycle^1–6^. The cumulative advantage hypothesis (SEP and health disadvantages interact and accumulate over time, indicating higher inequalities by age) and/or the age-as-leveler hypothesis (the inevitable decline of health dominates social differences at older ages) are the predominant explanations, and studies find evidence in favour of the former^1, 2^, the latter^6^, or both hypotheses^4^. Integrating a life-course perspective to explore associations at different ages, therefore, is important for understanding the pathways through which SEP gets “under the skin”^4^.

Systemic inflammation has been proposed as a physiological process linking SEP to health, and it can emerge though several pathways^7–10^; the more widely held of which are stress-mediated factors and psychosocial processes involving the hypothalamic axis and the sympathetic and parasympathetic nervous systems^8–11^. Inflammation, therefore, may be crucial in understanding the evolution of SEP inequalities in morbidity and mortality risks at different ages^12^.

Most existing studies do not explicitly consider the full adult age span when analysing the association between SEP and inflammation (often assessed using C-reactive protein (CRP) and fibrinogen biomarkers), they are limited to describing associations between measures of SEP at various life stages (such as childhood and adulthood) and the inflammatory biomarkers in adulthood^13–19^. In general, these studies have found a negative association between SEP and CRP and fibrinogen, which in some reports shows sex differences^17^; however it is unclear how these associations differ across the lifespan. Although the SEP-inflammation association has been hypothesized to vary by age^20, 21^, this has received limited attention in the literature, being explicitly explored in very few studies and revealing no consistent pattern. For example, studies in the USA and Finland found a negative SEP-CRP association for those aged less than 60 years but not for older age groups^20, 22^, while another US study found systematic (but diminishing with age) educational differences in CRP for those aged 50+^21^.

Over and above the psychosocial pathway (such as stress, affective states etc.), health-related lifestyle indicators (such as Body Mass Index (BMI), smoking, physical activity, and diet quality) have been found to be important pathways though which SEP might impact inflammation burden^8, 14, 16, 17, 23^, with BMI appeared to exert the most prominent role^8, 13, 14, 23^. However, no consensus has been found on the importance of their mediation roles^14, 17, 18, 23, 24^. Age could be also an important factor in understanding the extent to which these behaviours explain the SEP-inflammation association because age may moderate the association of health-related behaviours with both SEP^3^ and inflammation^25, 26^. Moreover, the effects of health-related lifestyle factors on the incidence of adverse health outcomes are often characterized by cumulative, long-lasting processes^27^. These indicate the importance of taking a life span perspective with respect to the relationships between SEP, health-related lifestyle indicators and inflammation.

The current study examines the association of two measures of SEP (education and household income) with inflammatory biomarkers (CRP and fibrinogen) across the adult age span using a large national representative sample (*Understanding Society*: the United Kingdom Household Longitudinal Study; UKHLS). We capitalize on the wide age range available to investigate how the association of SEP with CRP and fibrinogen varies by age, allowing for flexible associations between age, SEP and the inflammatory biomarkers. This is the first study that examines whether SEP inequalities in inflammatory markers are apparent across the full adult age span, and if not when they emerge and/or converge. Finally, we explore the role of health-related lifestyle indicators in attenuating the SEP-inflammation gradients across the age span.

## Results

From the study potential sample of 9,181 respondents (UKHLS, wave 2), 754 had missing CRP (including those with CRP > 10 mg/L) or fibrinogen values, 267 had no or invalid BMI data and 217 lacked information on the remaining variables, giving an analytical sample of 7,943 individuals. Comparisons between the raw means of the potential and analysis sample showed limited differences in the utilized variables, suggesting that the impact of item missingness might be negligible.

### Bivariate analysis across age groups

Table 1 presents the mean levels of CRP (Panel A) and fibrinogen (Panel B) by gender, household income (in tertiles), education and lifestyle indicators for different age groups and for the total sample. Mean CRP levels were lower for those belonging to higher versus lower socio-economic groups for both education and income markers of SEP in all age groups except the younger (25–34) and the older (80+). To allow for a clearer presentation of how the unadjusted associations of the inflammatory biomarkers with each of the variables used in our analysis varied by age groups, percentage differences in the inflammatory biomarkers by categories of these variables across age groups are also presented in Table 1. For example, differences in mean CRP levels between the lowest and the highest income tertiles reached a maximum of about 0.565 mg/L at the 35–49 age group (i.e., moving from the highest to the lowest income tertiles there is a 35.4% increase in the mean CRP levels). Similarly, the education gradient in CRP emerged in the 35–49 age group after which it peaked in the 50–64 age group (lowest/highest education group mean CRP differences of 0.931 mg/L, which corresponded to a 54.8% increase in CRP when switching from “degree” to “no qualifications”) and then began to narrow.

**Table 1:** Mean CRP and fibrinogen levels by gender, SEP and health-related lifestyle indicators across different ages^a^.

	Age groups					
	25–34 (n = 964)	35–49 (n = 2357)	50–64 (n = 2516)	65–79 (n = 1771)	80 + (n = 335)	Total sample (n = 7943)
**Panel A. Mean CRP (mg/L)**						
***Gender***						
Males	1.373	1.786	2.028	2.192	2.533	1.872
Females	1.962	1.997	2.254	2.459	2.390	2.163
% CRP difference [p-value for no differences]^b^	42.9% [0.000]	11.8% [0.009]	11.1% [0.005]	12.2% [0.008]	−5.6% [0.542]	15.5% [0.000]
p-value for between age groups equality^c^	0.005					
***Equivalised household income (in tertiles)***						
High household income	1.490	1.593	1.931	2.101	2.591	1.804
Middle household income	1.775	1.942	2.052	2.401	2.524	2.048
Low household income	1.704	2.158	2.449	2.490	2.254	2.213
% CRP difference [p-value for no differences]^b^	14.4% [0.147]	35.4% [0.000]	26.8% [0.000]	18.5% [0.004]	−13% [0.234]	22.7% [0.000]
p-value for between age groups equality^c^	0.000					
***Educational attainment***						
Degree	1.594	1.574	1.791	1.756	2.075	1.656
Other higher degree	1.804	2.001	2.035	2.085	2.430	2.009
A-level	1.521	1.921	2.100	2.356	3.159	1.952
O-level/basic qualifications	1.835	2.098	2.114	2.312	2.129	2.100
No qualifications	1.437	2.102	2.772	2.676	2.596	2.556
% CRP difference [p-value for no differences]^b^	−9.8% [0.539]	33.5% [0.000]	54.8% [0.000]	52.4% [0.000]	25.1% [0.454]	54.3% [0.000]
p-value for between age groups equality^c^	0.000					
***Body Mass Index***						
1^st^ tertile	1.245	1.172	1.398	1.519	1.858	1.328
2^nd^ tertile	1.532	1.807	1.899	2.212	2.759	1.917
3^rd^ tertile	2.625	2.714	2.947	3.020	2.725	2.837
% CRP difference [p-value for no differences]^b^	111% [0.000]	131% [0.000]	111% [0.000]	98.8% [0.000]	46.7% [0.001]	113.6% [0.000]
p-value for between age groups equality^c^	0.001					
***Smoking status***						
Current smokers	1.717	2.307	2.705	2.792	2.918	2.310
Ex-smokers	1.756	1.805	2.003	2.421	2.681	2.060
Never smokers	1.554	1.744	1.978	2.092	2.164	1.849
% CRP difference [p-value for no differences]^b^	−9.5% [0.218]	−24.4% [0.000]	−26.9% [0.000]	−25.1% [0.000]	−25.8% [0.060]	−20.0% [0.000]
p-value for between age groups equality^c^	0.000					
***Sports activities***						
Sports participation	1.642	1.732	1.894	1.947	2.542	1.789
No sports participation	1.685	2.098	2.314	2.531	2.435	2.231
% CRP difference [p-value for no differences]^b^	2.62% [0.734]	21.1% [0.000]	22.2% [0.000]	29.9% [0.000]	−4.21% [0.719]	24.71% [0.000]
p-value for between age groups equality^c^	0.002					
***# of days walking***						
0	1.837	2.138	2.527	2.701	2.506	2.349
1–5	1.671	1.760	1.971	2.297	2.654	1.913
5–15	1.434	1.859	2.053	2.204	2.215	1.875
16+	1.611	1.822	1.964	1.956	2.123	1.856
% CRP difference [p-value for no differences]^b^	−12.3% [0.097]	−14.8% [0.020]	−22.3% [0.000]	−27.6% [0.000]	−15.3% [0.296]	−21.0% [0.000]
p-value for between age groups equality^c^	0.090					
***Five fruits/vegetables per day***						
Reported	1.881	1.625	1.891	2.064	2.427	1.882
Not reported	1.618	1.963	2.227	2.441	2.462	2.061
% CRP difference [p-value for no differences]^b^	−14.0% [0.120]	20.8% [0.000]	17.8% [0.000]	18.3% [0.000]	1.4% [0.903]	9.5% [0.001]
p-value for between age groups equality^c^	0.003					
***Non-full fat milk***						
Reported	1.661	1.841	2.133	2.307	2.507	2.006
Not reported	1.637	2.246	2.234	2.534	2.182	2.126
% CRP difference [p-value for no differences]^b^	-1.4% [0.877]	22.0% [0.000]	4.7% [0.433]	9.8% [0.161]	-13% [0.308]	6% [0.060]
p-value for between age groups equality^c^	0.044					
***Non-White bread***						
Reported	1.705	1.754	2.036	2.184	2.340	1.934
Not reported	1.583	2.222	2.398	2.688	2.741	2.211
% CRP difference [p-value for no differences]^b^	-7.2% [0.319]	26.7% [0.000]	17.8% [0.000]	23.1% [0.000]	17.1% [0.118]	14.3% [0.000]
p-value for between age groups equality^c^	0.000					
**Panel B. Mean fibrinogen (g/L)**						
***Gender***						
Males	2.368	2.613	2.733	2.883	2.981	2.659
Females	2.574	2.675	2.857	2.971	3.092	2.780
% fibrinogen difference [p-value for no differences]^b^	8.7% [0.000]	2.4% [0.005]	4.5% [0.000]	3.1% [0.000]	3.7% [0.054]	4.6% [0.000]
p-value for between age groups equality^c^	0.000					
***Equivalised household income (in tertiles)***						
High household income	2.380	2.555	2.738	2.881	3.020	2.651
Middle household income	2.479	2.664	2.792	2.938	3.055	2.730
Low household income	2.541	2.718	2.860	2.968	3.048	2.783
% fibrinogen difference [p-value for no differences]^b^	6.8% [0.000]	6.4% [0.000]	4.5% [0.000]	3.5% [0.015]	0.93% [0.863]	5.0% [0.000]
p-value for between age groups equality ^c^	0.040					
***Educational attainment***						
Degree	2.431	2.531	2.685	2.837	2.986	2.573
Other higher degree	2.458	2.630	2.784	2.836	2.978	2.695
A-level	2.433	2.667	2.795	2.918	2.999	2.681
O-level/basic qualifications	2.532	2.713	2.810	2.944	3.017	2.768
No qualifications	2.608	2.800	2.921	2.994	3.091	2.940
% fibrinogen difference [p-value for no differences]^b^	7.3% [0.002]	10.6% [0.000]	8.8% [0.000]	5.5% [0.000]	3.5% [0.111]	14.3% [0.000]
p-value for between age groups equality^c^	0.109					
***Body Mass Index***						
1^st^ tertile	2.350	2.486	2.688	2.860	3.029	2.577
2^nd^ tertile	2.438	2.607	2.758	2.891	3.086	2.700
3^rd^ tertile	2.736	2.844	2.917	3.014	2.984	2.891
% fibrinogen difference [p-value for no differences]^b^	16.4% [0.000]	14.4% [0.000]	8.5% [0.000]	5.4% [0.000]	-1.5% [0.622]	12.2% [0.000]
p-value for between age groups equality^c^	0.000					
***Smoking status***						
Current smokers	2.505	2.788	3.001	3.146	3.284	2.810
Ex-smokers	2.474	2.613	2.720	2.906	3.006	2.719
Never smokers	2.436	2.592	2.758	2.890	3.049	2.679
% fibrinogen difference [p-value for no differences]^b^	−2.8% [0.053]	−7.0% [0.000]	−8.1% [0.000]	−8.1% [0.000]	−7.2% [0.557]	−4.7% [0.000]
p-value for between age groups equality^c^	0.000					
***Sports activities***						
Sports participation	2.463	2.601	2.722	2.850	2.982	2.632
No sports participation	2.474	2.700	2.848	2.970	3.055	2.802
% fibrinogen difference [p-value for no differences]^b^	0.4% [0.736]	3.8% [0.000]	4.6% [0.000]	4.2% [0.000]	2.4% [0.309]	6.5% [0.000]
p-value for between age groups equality^c^	0.015					
***# of days walking***						
0	2.494	2.699	2.865	3.009	3.090	2.814
1–5	2.439	2.614	2.779	2.917	2.993	2.681
5–15	2.446	2.625	2.782	2.904	2.786	2.674
16+	2.482	2.647	2.755	2.857	2.989	2.694
% fibrinogen difference [p-value for no differences]^b^	−0.5% [0.860]	−1.9% [0.155]	−3.8% [0.000]	−5.1% [0.000]	−3.3% [0.021]	−4.3% [0.000]
p-value for between age groups equality^c^	0.106					
***Five or more fruits/vegetables per day***						
Reported	2.441	2.609	2.763	2.903	3.007	2.723
Not reported	2.472	2.654	2.808	2.940	3.050	2.721
% fibrinogen difference [p-value for no differences]^b^	1.3% [0.471]	1.7% [0.107]	1.6% [0.050]	1.3% [0.182]	1.4% [0.537]	-0.1% [0.926]
p-value for between age groups equality^c^	0.997					
***Non-full fat milk***						
Reported	2.463	2.637	2.796	2.932	3.046	2.722
Not reported	2.486	2.699	2.806	2.905	3.018	2.717
% fibrinogen difference [p-value for no differences]^b^	0.9% [0.577]	2.4% [0.049]	0.4% [0.748]	-0.9% [0.505]	-0.9% [0.724]	-0.2% [0.744]
p-value for between age groups equality^c^	0.471					
***Non-white bread***						
Reported	2.482	2.605	2.783	2.911	3.018	2.700
Not reported	2.445	2.736	2.830	2.973	3.100	2.744
% fibrinogen difference [p-value for no differences]^b^	−1.5% [0.245]	5% [0.000]	1.7% [0.036]	2.1% [0.025]	2.7% [0.183]	1.7% [0.012]
p-value for between age groups equality^c^	0.000					

^a^Age groups selected to reflect different life stages. Bivariate analyses were adjusted to account for sample weights.

^b^Percentage difference in CRP and fibrinogen levels were calculated comparing the mean biomarker levels between gender, SEP and health-related lifestyle indicators. The lowest and the highest categories were used for the case of these variables that had more than two categories and expressed changes as a percentage of the mean CRP or fibrinogen levels of the first -as presented in the table- category of each variable. P-values for absence of differences in CRP and Fibrinogen levels by gender, SEP groups and ealth-related lifestyle indicators are calculated using chi-squared two-tailed tests (for each of the age groups and for the whole sample).

^c^The presence of systematic heterogeneity in the association between CRP/fibrinogen and the other variables (gender, SEP measures and lifestyle indicators) across age groups was tested by two-tailed F-tests for the joint significance of interaction terms of the (categories of the) latter variables with age categories.

Lower fibrinogen levels were apparent for those with higher income or educational attainment. However, unlike CRP, statistically significant age variations in the SEP-fibrinogen association were only evident when household income was used as a SEP marker (two-tailed p-value for between age groups equality = 0.040). Systematic bivariate associations were also found between the health-related lifestyle indicators and the inflammatory biomarkers (Table 1); in most cases, these associations seem to vary across the age groups.

### Association of SEP with CRP and fibrinogen

Table 2 shows the estimation results (OLS and GLM models), predicting the mean CRP and fibrinogen levels by household income tertiles and educational attainment, after adjustments for age (cubic polynomials), gender and regional indicators (“base model”). There were statistically significant differences in inflammation for both SEP measures examined (p-values for trend: 0.000). Specifically, both CRP and fibrinogen levels monotonically increased with decreasing household income (in tertiles). For example, the predicted mean CRP levels for those at the lower versus the higher tertile of the household income distribution were 2.192 mg/L (95% confidence interval [CI]: 2.102, 2.282) versus 1.819 mg/L (95% CI: 1.737, 1.902). This indicated a difference in CRP levels of about 0.373 mg/L (95% CI: 0.249, 0.496). The corresponding income differences in fibrinogen were about 0.124 g/L (95% CI: 0.092, 0.156).

**Table 2:** Predicted C-reactive protein (mg/L) and fibrinogen (g/L) levels by household income and education.

	C-reactive protein (mg/L)		Fibrinogen (g/L)	
	Mean	95% CI	Mean	95% CI
***Equivalised net HH income***				
High HH income	1.819	1.737; 1.902	2.654	2.632; 2.676
Middle HH income	2.051	1.964; 2.137	2.733	2.711; 2.755
Low HH income	2.192	2.102; 2.282	2.778	2.756; 2.801
Differences as % of the biomarker’s s.d.^a^	20.7%		23%	
P-value _trend_	0.000		0.000	
***Education***				
Degree	1.707	1.611; 1.802	2.626	2.601; 2.651
Other higher degree	1.996	1.855; 2.136	2.686	2.651; 2.722
A-level	2.022	1.900; 2.143	2.730	2.699; 2.762
O-level/other qualification	2.091	1.996; 2.186	2.759	2.735; 2.783
No qualification	2.379	2.247; 2.512	2.821	2.786; 2.856
Differences as % of the biomarker’s s.d.^a^	33.7%		36.2%	
P-value_trend_	0.000		0.000	

Abbreviations: CI, confidence interval; HH, household; s.d., standard deviation.

Notes: Predicted values were obtained from OLS models (fibrinogen) and GLMs (CRP) adjusted for age, gender, region and urbanization indicators (“base model”). P-values_trend_ (tests for trend in the mean values of CRP and Fibrinogen across SEP categories) were obtained by entering the SEP measures as continuous variable in the models.

^a^Predicted differences in the mean CRP and fibrinogen levels between the lowest and the highest SEP groups were expressed as percentages of the standard deviation of the corresponding inflammatory biomarker.

On average, the predicted CRP levels were higher by about 0.672 mg/L (95% CI: 0.505, 0.840) for those with no education qualifications compared to those with a degree. The corresponding education differences in fibrinogen were about 0.195 g/L (95% CI: 0.151, 0.239). The SEP differences were comparable in magnitude between CRP and fibrinogen for each different SEP measure as results expressed as percentage of the biomarkers’ standard deviations show.

### Heterogeneity by Age

**Figure 1:**
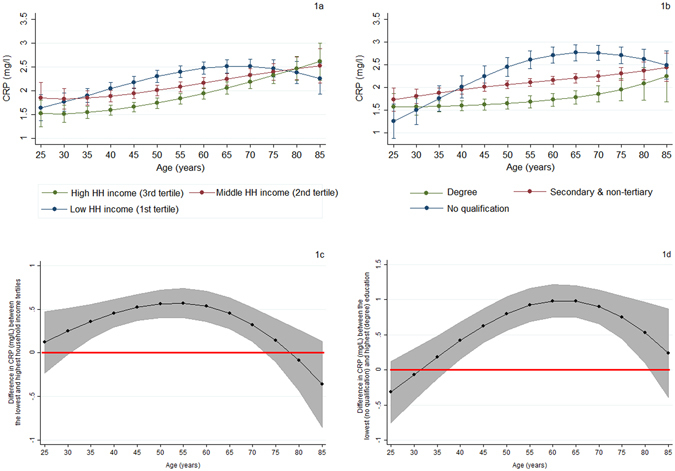
Trajectories of CRP (mg/L) and the predicted CRP gradients by household income and education across age. (**a**) Trajectories of CRP by equivalised net household income (capped spikes: 95% confidence intervals); (**b**) Trajectories of CRP by education (capped spikes: 95% confidence intervals); (**c**) Differences (in absolute terms) in predicted CRP levels between the lowest and highest household income tertiles (shaded area: 95% confidence intervals); (**d**) Differences (in absolute terms) in predicted CRP levels between the lowest and highest education category (shaded area: 95% confidence intervals).

**Figure 2:**
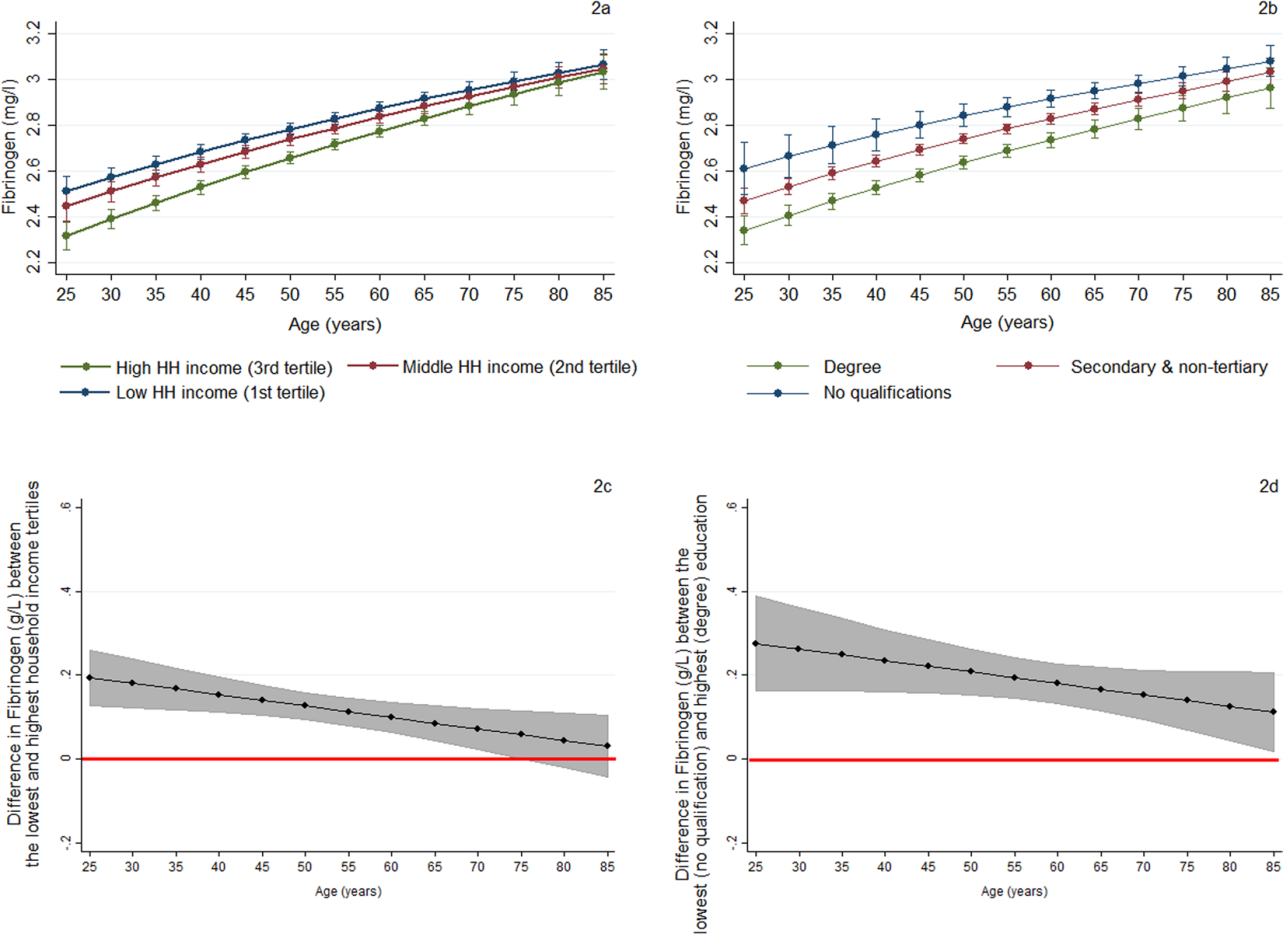
Trajectories of fibrinogen (g/L) and the predicted fibrinogen gradient by household income and education across age. (**a**) Trajectories of fibrinogen by equivalised net household income (capped spikes: 95% confidence intervals); (**b**) Trajectories of fibrinogen by education (capped spikes: 95% confidence intervals); (**c**) Differences (in absolute terms) in predicted fibrinogen levels between the lowest and highest household income tertiles (shaded area: 95% confidence intervals); (**d**) Differences (in absolute terms) in fibrinogen between the lowest and highest education category (shaded area: 95% confidence intervals).

Figure 1a and b depict the predicted age trajectories of CRP by household income and education groups (range plots with capped spikes: 95% CI), obtained by adding SEP-age polynomial interactions to the base models (Table 2). In order to simplify the visualisation of the education differences in inflammation by age (Figs 1b and 2b), we group the middle three education categories into one, while for the rest of our analysis the detailed set of education categories was employed; the corresponding graphs in the case of the detailed education categories revealed practically identical patterns to those in Figs 1b and 2b (see Supplementary Fig. S1). To facilitate interpretation, the corresponding absolute differences in CRP levels between the lowest and the highest SEP groups (i.e., income and education gradients in CRP) are also presented (Fig. 1c and d). Figure 1a and b show increasing predicted CRP levels with increasing age for all SEP groups. Those with lower SEP have higher CRP levels compared to those of higher SEP across most of the age groups; except young adulthood and very old ages where non-statistically significant SEP differences in CRP were observed (chi-squared tests: two-tailed p-values > 0.05). Figure 1c and d further illustrate the presence of age heterogeneity in SEP differences in CRP (SEP-age interactions: p-values < 0.001). Specifically, income-related inequalities in CRP emerged around the age of 30 (zero lies outside the 95% CI), after which they peaked at the age of 55 (difference between lowest/highest income group: 0.569 mg/L; 95% CI: 0.400, 0.737) and then began to narrow, converging around the age of 75. The education gradient in CRP emerged and peaked slightly latter (around the age of 37 and 60 years, respectively); the largest education difference in CRP is about 0.983 mg/L (95% CI: 0.752, 1.215). This gradient then reduced with age and became non-significant at around the age of 80 years.

Figure 2 presents the corresponding SEP differences in fibrinogen. Mean fibrinogen levels increase with age across all SEP groups (Fig. 2a and b). The income gradients in fibrinogen decrease with age as illustrated by Fig. 2c (income-age interactions; two-tailed p-value = 0.033). On the other hand, no systematic age variations were evident for the education gradients (Fig. 2d; education-age interactions: two-tailed p-value > 0.10).

### Examining the role of health-related lifestyle factors: BMI, healthy diet, smoking status and physical activity

**Figure 3:**
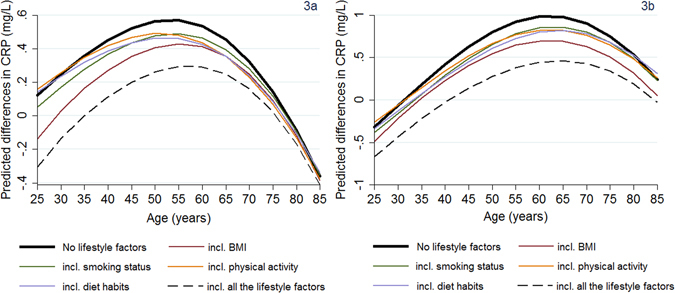
Predicted SEP gradient in CRP (mg/L) by age: the role of ΒΜΙ, healthy diet, smoking and physical activity. (**a**) Presents (absolute) predicted differences in the mean CRP levels between the lowest and the highest tertile of the household income distribution (income gradients in CRP) obtained with or without accounting for the health-related lifestyle indicators. (**b**) Presents the corresponding differences in CRP between those with no education qualifications compared to those with a degree.

Figure 3 presents CRP differences between the lowest and the highest SEP groups across different ages before (identical to Fig. 1c and d) and after adjustments for health-related lifestyle indicators (either separately or concurrently). The fact that the CRP differences by SEP reduced in magnitude after adjusting for lifestyle factors suggested that each of these lifestyle factors partially (but not fully) attenuated the SEP gradients in CRP (F-test for the joint significance of SEP main effects and their age interactions across models accounting for lifestyle indicators; p-values = 0.000). BMI played the greatest attenuating role. The SEP gradients in CRP remained apparent even accounting for all the lifestyle factors jointly (p-values = 0.000).

Notable age variations were observed in the SEP differences in CRP that attributed to the role of each lifestyle factor (i.e., health behaviour pathways; obtained by comparing the SEP gradient with and without accounting for each of the lifestyle indicators at different ages). Specifically, accounting for BMI or smoking resulted in lower reductions in the income gradients in CRP with increasing age (Fig. 3a). For example, following adjustments for BMI, the difference in the CRP levels between the lowest and the highest income group (income gradient in CRP) reduced by about 55% at the age of 35 (from 0.359 mg/L to 0.165 mg/L; Fig. 3a), while the corresponding reduction was 22% at the age of 70 (from 0.322 mg/L to 0.247 mg/L; Fig. 3a). On the other hand, accounting for physical activity or healthy diet habits, a higher reduction in the income gradients in CRP was evident at early and later middle life rather than at younger ages (Fig. 3a).

BMI played the greatest role in attenuating the education gradients in CRP (Fig. 3b). Focusing at the age interval for which education differences in CRP are evident (i.e., 40s to late 70s; Fig. 1d), we found limited age variations in the attenuating roles (i.e. percentage of the SEP gradient explained) of BMI and physical activity. Following BMI adjustments, the education gradients in CRP reduced by about 35% vs 30% at the age of 40 and 70, respectively. The attenuating roles of smoking and healthy diet habits were larger at early and later middle life compared to older ages (Fig. 3b). For example, smoking status accounted for 40% of the education gradients in CRP at the age of 40 compared to 9% at around the age of 75; similar in magnitude was the attenuating role of our healthy diet indicators (i.e., accounted for about 35% and 9% of the education gradients in CRP at the age of 40 and 75, respectively).

**Figure 4:**
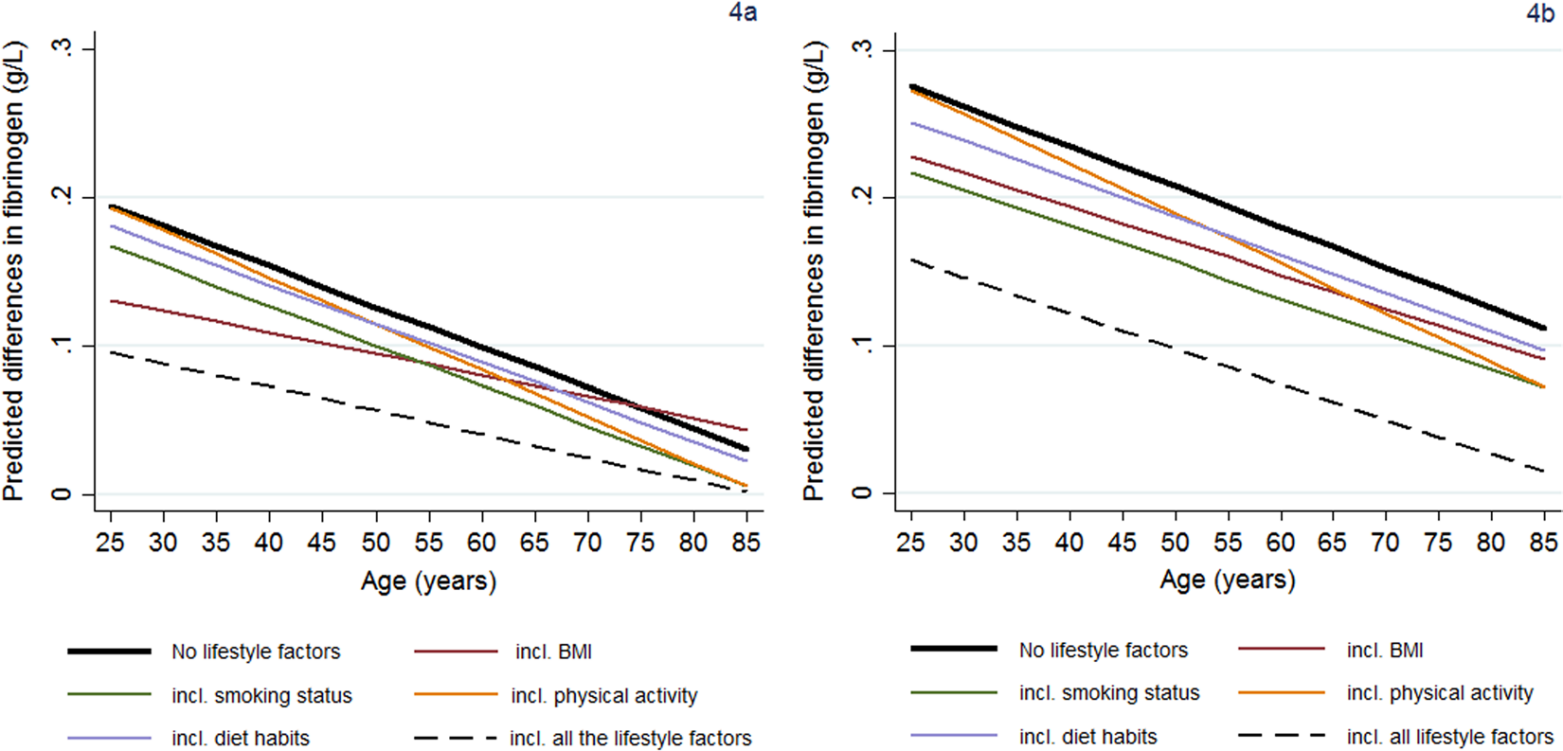
Predicted SEP gradients in fibrinogen (g/L) by age: the role of ΒΜΙ, healthy diet, smoking and physical activity. (**a**) Presents (absolute) predicted differences in the mean fibrinogen levels between the lowest and the highest tertile of the household income distribution (income gradients in fibrinogen) obtained with or without accounting for the health-related lifestyle indicators. (**b**) Presents the corresponding differences in fibrinogen between those with no education qualifications compared to those with a degree.

The income gradients in fibrinogen are presented in Fig. 4a. BMI became less relevant in attenuating the income differences in fibrinogen as age increases, while the opposite holds for physical activity (Fig. 4a). For example, 33% of the income differences in fibrinogen are attributed to the role of BMI at the age of 25 versus 8% at the age of 70. Smoking seemed to explain a larger (percentage) part of the income gradients in fibrinogen by age; smoking adjustments reduced the income gradient in fibrinogen by about 15% (from 0.194 g/L to 0.167 g/L; Fig. 4a) around the age of 25 versus 35% (from 0.072 g/L to 0.046 g/L; Fig. 4a) for those at 70 s. On the other hand, there were limited age variations in the attenuating role of healthy diet in the income gradient in fibrinogen. Simultaneously accounting for all the lifestyle indicators resulted in reduced, but still evident income gradient in fibrinogen (f-test for the joint significance of income main effects and their age interactions; two-tailed p-value = 0.000). The results for education are presented in Fig. 4b; however, as had been already stated, the education gradients in fibrinogen did not systematically vary by age (education-age interactions; two-tailed p-values > 0.10).

### Sensitivity analysis

Sensitivity analysis with medications resulted in practically identical results compared to those presented in the main analysis. Specifically, accounting for medications did not change the observed inequality patterns by age or the explanatory role of each of the health-related lifestyle indicators on the pertinent inequalities. Moreover, our finding did not substantially differ after excluding people taking medications that may affect CRP or fibrinogen levels (about 18% of the sample) from the analysis, suggesting that our conclusions are not subject to potential biases associated with the role of medications.

## Discussion

Using data from a large nationally representative sample of adults aged 25 and older (UKHLS), we find that the negative association between higher SEP (assessed by education attainment and equivalised household income) and inflammatory biomarkers (CRP and fibrinogen) follows heterogeneous patterns as a function of age. These age patterns vary by the inflammatory biomarker examined (CRP or fibrinogen) rather than the utilised marker of SEP. In general, we find that SEP inequalities in CRP emerge in people aged in their 30 s, and increase up to mid-50s or early 60 s, when they peaked. They then begin to narrow, converging at older ages (75 and 85 for income- and education-related inequalities, respectively). The SEP inequalities in fibrinogen monotonically decrease with age; however, these inequalities systematically vary with age only when income is used as an SEP marker.

Accounting for BMI, smoking status, physical activity and healthy diet habits - either separately or jointly - explains part (but not all) of SEP gradients in CRP as a function of age. In general, BMI exerted the largest explanatory role compared with other health-related lifestyle indicators. We find considerable age heterogeneity in the SEP differences in inflammation that attributed to the role of each lifestyle indicator; these age variations are specific to both the inflammatory biomarker and the SEP measure considered. Most of the lifestyle indicators either become gradually less relevant in attenuating the magnitude of the SEP differences in inflammation with increasing age or exerts attenuating roles that accumulate with age, becoming more evident at early and later middle life than at younger ages.

Allowing for flexibility in the negative association between SEP and inflammatory biomarkers with age, this study demonstrated strong age-specific patterns that have often been masked in the previous literature^13–19^. In relation to age heterogeneity in SEP-CRP association, our findings are broadly consistent with the few studies available that suggest negative associations in individuals younger than 60 but not in older age groups^20, 22^. A US study on people aged 50+ have found that the educational differences in CRP diminish with age^21^. Our evidence regarding reduced income-inequalities in fibrinogen by age extends the limited number of previous studies that have compared similar associations across arbitrarily defined age groups^20^, to a representative Great Britain sample of the whole age range.

In general, these findings highlight that no single hypothesis explains the observed age patterns in the SEP inequalities in inflammation. Features from both the cumulative advantage and the age-as-leveler hypotheses can be integrated in a dynamic relationship, with their relative importance varying across the lifespan. Specifically, the fact that SEP inequalities in CRP are concave with age, peaking in later middle life, is consistent with the cumulative advantage hypothesis until midlife, and the age-as-leveler hypothesis may be the leading driving force thereafter. However, this finding is not universal because the age patterns associated with SEP inequalities in fibrinogen appear to imply the dominant role of the age-as-leveler over the cumulative advantage hypothesis across the lifespan. This indicates that deterioration in health as an inevitable biological process (age-as-leveler hypotheses) is more relevant for SEP association across the whole adult lifespan on fibrinogen than CRP; perhaps because CRP purely reflects inflammatory load that may accumulate and interact over time, while fibrinogen also reflects other physiological processes such as haemostasis^28^.

The fact that health-related lifestyle indicators partially but not fully attenuate SEP inequalities in inflammation is consistent with several previous studies^8, 14, 16, 17, 23^ and indicates that although the health behaviours are important pathways in the SEP-inflammation association, additional mechanisms – such as stress-related physiological responses to disadvantaged SEP – may be part of the process. Moreover, the dominant role of BMI in explaining these inequalities is not unexpected^13, 14, 23^; excess adiposity is strongly associated with elevated CRP ^14^ and is socially patterned^29^. Of particular importance, our results about the prominent role of BMI -as opposed to all the other lifestyle factors considered (smoking, physical activity and diet quality)- are confirmed by a recent SEP-inflammation study, where a wider array of inflammatory markers and a more comprehensive diet quality measure was examined^23^. Our findings that the explanatory role of these lifestyle indicators is age specific and varies by the measure of the SEP may reflect disparities in the association between the lifestyle indicators and both inflammation^25, 26^ and SEP^3^ across different ages. However, our results imply that health promotion policies aimed toward supporting disadvantaged people in adopting healthy behaviours at younger ages may be beneficial in both the short and long term. This is because accounting for health-related lifestyle indicators resulted in substantial attenuations in the magnitude of the SEP inequalities in inflammation at young adulthood, or their health impacts may accumulate and thus be more effective later in life.

In accordance with some^9, 24, 30–32^, but not all^14, 17^, of the existing reports, our findings indicated that there was no moderating role of sex on the association between SEP and inflammation. These disparities may be attributed to differences in both age ranges and population composition across different studies. For instance, although some US studies have suggested gender differences^14, 17^, such studies were limited to a narrow age range and their findings were not always confirmed by evidence from the UK^33^.

This paper is the first to provide a thorough examination of SEP inequalities in CRP and fibrinogen allowing for flexible age gradients as well as both current (household income) and more distal (education) SEP measures. However, there are a number of potential limitations. Firstly, the analyses are broadly cross-sectional in nature and, thus, we are not able to make assertions about causality or to disentangle age and cohort effects. However, van Kippersluis *et al*. found no consistent cohort effects on income-related inequalities in health for the UK^5^. Although further longitudinal research is needed to elucidate these issues, our study highlights the importance of considering a wide age span in the case of the SEP inequalities in health. Secondly, our inflammatory biomarkers do not capture all aspects of inflammation given the complexity of the inflammatory profiles and their regulation (such as synergistic effects of inflammation biomarkers or the resolving role of anti-inflammatory mechanisms)^23, 34, 35^. For example, CRP captures inflammatory load and therefore does not provide information of specific pro- and anti-inflammatory pathways or other aspects of immunity. However, more specific biomarkers such as the interleukins, other cytokines, interferons or complement factors were not available in UKHLS. Our results seemed to be broadly in line with studies that examine a wider range of measures of inflammation^23^ and further research is needed to confirm the generalisability of the proposed age patterns in the case of a broader set of inflammatory biomarkers. A further concern is that smoking status, physical activity and adoption of healthy diet habits are based on self-reported data which may be subject to measurement error. Moreover, even though similar diet variables were used in previous SEP-inflammation studies^33^, our diet indicators are only partial markers of diet quality. Future research that allows for more detailed diet information may be useful to better understand its role on shaping the SEP inequalities in inflammation across the life-cycle. Thirdly, our sample, while representative of the GB population, is predominantly composed of White/European participants and therefore we cannot generalize to non-White groups. Studies suggesting ethnic differences in SEP-inflammation associations originate from the US^14, 17, 21^ and may not apply to the UK ethnic minority groups. Fourthly, our results may be biased because of selective dropout and exclusion of those on anti-inflammatory medications at blood sample collection. However, sample weights account for these unequal selection effects. Finally, mortality selection may distort associations at older ages, although it has been shown that needs not necessarily be true^36^. Mortality rates in 2013 in the UK did not exceed 2% before the age of 72 years and 5% before the age of 80 years^37^; this may indicate that our results for those 80+ should be treated with caution.

By focusing on inflammatory biomarkers, which have been proposed as a physiological process linking SEP to health, we highlight the importance of considering a life span perspective in order to better understand the process through which SEP gets “under the skin”. We find that SEP inequalities in inflammation are concave or diminish with age, depending on the inflammatory biomarker considered. These results may indicate the importance of integrating features from both existing life-course theories (cumulative advantage and the age-as-leveler hypothesis), with their relative role varying across the lifespan, in order to explain the observed heterogeneity in the SEP inequalities in health across the life span. The attenuating role of health-related lifestyle indicators suggests that targeting health promotion policies may help to reduce SEP inequalities in health.

## Methods

### Data source and sample

This study used data from the General Population Sample (GPS) of the UKHLS, which is a large, national representative panel survey covering about 32,000 households in the UK at wave 1^38^. For this analysis, we focus on GPS respondents who took part in a nurse health assessment (NHA) interview, which was conducted five months on average after the wave 2 main survey. Respondents were eligible for the NHA interview if they took part in the main wave 2 survey, were aged 16+, lived in Great Britain (not Northern Ireland), conducted their interview in English, and were not pregnant.

At wave 2 (1/2010–3/2012) the GPS had a response rate of 80% of households invited to take part, and within them 90% of individuals participated^38^. From the 26,961 respondents who were eligible for the NHA interview at wave 2, 15,591 took part.

Α blood sample was collected from unfasted participants during the NHA interview, provided the respondent had no clotting or bleeding disorder and had never had a fit or taking anti-clotting medications (eligible sample: 14,264). Of these, 10,782 consented to the blood collection, and for 9,803 of those data for at least one biomarker are available. We restricted our sample to individuals aged 25+ to focus on adults who are more likely to have completed their full-time education and be earning their own income^3, 39^, resulted in a potential sample of 9,181 respondents.

Participants gave informed written consent for their blood to be taken and stored for future scientific analysis. The UKHLS has been approved by the University of Essex Ethics Committee and the nurse data collection by the National Research Ethics Service (10/H0604/2). All methods were carried out in accordance with the approved guidelines and regulations.

### Measures

#### CRP and Fibrinogen

High-sensitivity CRP was measured from serum samples by the Haematology Department of the Royal Victoria Infirmary in Newcastle, using the N Latex CRP mono Immunoassay on the Behring Nephelometer II Analyzer. The intra and inter-assay coefficients of variation were below 2%^40^. Where CRP measurements were below the analysers’ detection limits (0.2 mg/L), values recoded to the half of the distance between zero and the detection limit (202 cases). Participants with CRP over 10 mg/L are excluded from analysis (523 cases), since this is considered evidence of current infection rather than chronic processes^41^.

Fibrinogen was measured from citrated serum using a modified Clauss thrombin clotting method on the IL-ACS-TOPS analyser. The intra and inter-assay coefficients of variation were less than 7%. The lower detection limit of the assay was 0.5 g/L^40^.

### Socio-economic position

Both equivalised net monthly household income (in tertiles) and educational attainment (collected at wave 2 main survey) were used to test the robustness of the results to different SEP measures^2–4^. Family income is a measure of the current SEP, while education is determined earlier in life and, thus, may help diminish simultaneity concerns and the role of any potential effects of health shocks on SEP^4^.

Income from all sources was collected from each household member and, if missing, was imputed (available as a derived variable)^38^. For the purpose of our analysis, this variable was equivalised (using the modified OECD scale) to adjust for household size and composition. Following previous literature, age-specific income tertiles were then created, for each 5-years age group^2–4^. Educational attainment captures the highest qualification achieved (degree, other higher degree, a-level, o-level/basic qualifications and no-qualifications).

### Health-related lifestyle indicators

Body weight and height measurements were measured by a nurse using a portable stadiometer and a digital floor body fat monitor/scale (Tanita BF 522). Respondents were required to remove their socks, shoes and any bulky clothing^42^. BMI was calculated as the weight (kilograms) over the square of height (meters). Smoking status was based on self-report data; respondents were classified as never smokers, current smokers and ex-smokers. Physical activity was proxied by sports activities (binary variable for engaged in sports at least monthly) and walking (a continuous variable for the number of days walking more than 30 minutes in the past four weeks). Finally, in accordance with previous studies^33^, healthy diet habits were proxied by consumption of fruits/vegetables (binary variable for consuming 5+ portions of fruits/vegetables per day^43^) and two dichotomous variables for using non-full fat (versus full fat) milk and non-white (versus white) bread.

### Other covariates

Age and gender were accounted for to capture demographic characteristics. We defined age as a continuous variable and it was mean-centred to reduce multicollinearity between age polynomials in the later statistical analysis^2^. Region (9 regions of England, Wales and Scotland) and urbanization indicators (urban or rural) were also incorporated to account for regional variations in health^44^.

### Statistical analysis

All analyses were weighted using probability sample weights to make the sample representative to the GB population. They were calculated by adjusting published weights for the wave 2 NHA^42^ to further account for blood data availability (mainly capturing consent and successful blood sample collection), using backward stepwise logistic regressions on predictors from the nurse visit.

Bivariate analyses between each of the inflammatory biomarkers and gender, SEP measures and health-related lifestyle indicators across different age groups as well as for the whole sample were implemented. For this descriptive analysis, BMI and days walking were categorized into tertiles and quartiles, respectively; however, for the rest of the analysis, they were employed as continuous variables.

Multivariate analyses were implemented to explore the association between inflammation and SEP measures after adjusting for covariates. Since CRP was characterized by a strongly skewed distribution, generalized linear models (GLMs) were estimated^45^. We employed the modified Park Test to determine the proper choice of the conditional variance function for the GLMs, and the Hosmer–Lemeshow Test to confirm that choice of link function provided the best fit to the data^45^. This analysis indicated that, among several alternative GLMs that typically used in case of skewed distributed data, the Poisson GLM with log link was the most appropriate for CRP. On the other hand, OLS regression models were used for fibrinogen because its distribution was approximated by a normal distribution. All regression models were estimated using heteroscedasticity robust standard errors^46^.

The GLMs for CRP and the OLS regressions for fibrinogen were estimated on the pooled sample of men and women (SEP-gender interactions; two-tailed p-values > 0.05). The “base models” accounted for demographic, regional variables and each of the SEP measures. In order to allow for a flexible association between the inflammatory biomarkers and age, different functions of age were tested. A cubic age polynomial provided the best statistical fit, and was included in the models and interacted with gender in order to capture gender differences in the age trajectories of these biomarkers.

To explore if the association of SEP with CRP and fibrinogen varied with age, the polynomial age variables were interacted with SEP (“age interacted base models”). The obtained age trajectories of CRP and fibrinogen by SEP, along with the corresponding SEP gradients (predicted differences in the biomarkers between the highest/lowest SEP) by age, are presented graphically.

In subsequent analysis, the extent to which these SEP gradients in CRP and fibrinogen were explained by the health-related lifestyle indicators across different ages was examined. For this reason, BMI, smoking status, physical activity and healthy diet measures were added to the models separately (to explore the attenuating role of each lifestyle factor) and jointly. Interactions of these lifestyle factors with age (or age polynomials) were also included in the models (when they were statistically significant) allowing for age flexibility on the pertinent associations. This analysis allowed for investigation of the SEP differences in inflammation that attributed to the role of each lifestyle factor (i.e., health behaviour pathways) and how these may vary by age.

As a sensitivity analysis, we examined the influence of statins and anti-inflammatory medications on our results by: a) including relevant dummies as covariates and b) excluding those taking these medications from the analysis.

### Data availability

The datasets analysed during the current study are available in the UK Data Archive (http://doi.org/10.5255/UKDA-SN-6614-9 and http://doi.org/10.5255/UKDA-SN-7251-3).

## Electronic supplementary material

Supplementary graphs
