# Supplementary material for: Socio-economic inequalities in C-reactive protein and fibrinogen across the adult age span: Findings from Understanding Society
Source: Sci Rep. 2017 Jun 1;7:2641. doi: 10.1038/s41598-017-02888-6 (PMC5454021; doi:10.1038/s41598-017-02888-6)

Socio-economic inequalities in C-reactive protein and fibrinogen across the adult age span: Findings from *Understanding Society*

Apostolos Davillas [1,*]; Michaela Benzeval[1]; Meena Kumari [1,2]

[1] Institute for Social and Economic Research (ISER), University of Essex, Colchester, UK

[2] Department of Epidemiology and Public Health, University College London, London, UK

Supplemental Material

Supplementary Figure S1. Trajectories of CRP (mg/L) and fibrinogen (mg/L) by the full set of education categories across age.

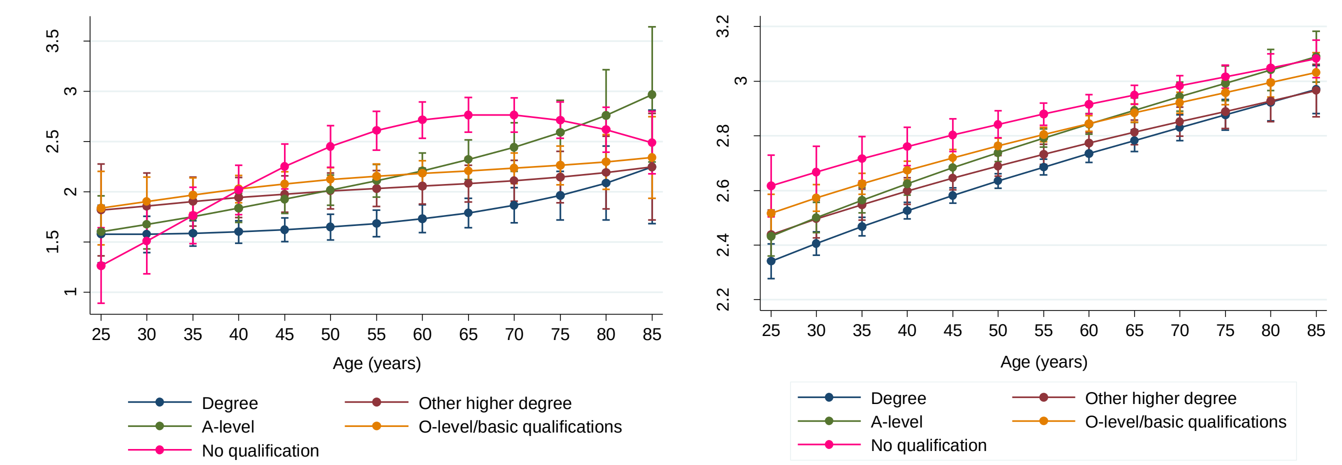

Supplement: Supplementary file 1 — Supplementary graphs [file 41598_2017_2888_MOESM1_ESM.pdf]
